# Electro-Haptic Enhancement of Spatial Hearing in Cochlear Implant Users

**DOI:** 10.1038/s41598-020-58503-8

**Published:** 2020-01-31

**Authors:** Mark D. Fletcher, Robyn O. Cunningham, Sean R. Mills

**Affiliations:** Faculty of Engineering and Physical Sciences, University of Southampton, University Road, Southampton, SO17 1BJ United Kingdom

**Keywords:** Auditory system, Translational research

## Abstract

Cochlear implants (CIs) have enabled hundreds of thousands of profoundly hearing-impaired people to perceive sounds by electrically stimulating the auditory nerve. However, CI users are often very poor at locating sounds, which leads to impaired sound segregation and threat detection. We provided missing spatial hearing cues through haptic stimulation to augment the electrical CI signal. We found that this “electro-haptic” stimulation dramatically improved sound localisation. Furthermore, participants were able to effectively integrate spatial information transmitted through these two senses, performing better with combined audio and haptic stimulation than with either alone. Our haptic signal was presented to the wrists and could readily be delivered by a low-cost wearable device. This approach could provide a non-invasive means of improving outcomes for the vast majority of CI users who have only one implant, without the expense and risk of a second implantation.

## Introduction

Cochlear implants (CIs) are neural prostheses that enable profoundly hearing-impaired people to perceive sounds through electrical stimulation of the auditory nerve. The CI is one of the greatest achievements of modern medicine. However, recent decades have not been marked by the huge improvements in CI technology that were seen in the 1980s and 1990s^1^, and CIs still have significant limitations^2–4^. One of the primary limitations of CIs is that users often struggle to locate and segregate sounds^5^. This leads to impaired threat detection and an inability to separate sound sources in complex acoustic scenes, such as schools, cafes, and busy workplaces. In normal-hearing individuals, the origin of a sound is determined by exploiting differences in the intensity and arrival time of sounds between the ears (interaural level and time differences), as well as by the direction-dependent spectral filtering of sounds by the pinnae. CI users have limited access to interaural level difference (ILD) and interaural time difference (ITD) cues, particularly the around 95% of users that are implanted only in one ear^6^. Furthermore, because of the poor spectral resolution of CIs^1^ and the fact that CI microphones are typically mounted behind the ear, CI users often have severely limited access to important spatial information usually given by the pinnae. We propose a new approach for enhancing spatial hearing in CI users by providing missing spatial hearing cues through haptic stimulation of the wrists.

There are several existing approaches for improving spatial hearing in CI users, although each has substantial limitations. For example, preservation of residual low-frequency acoustic hearing after implantation can give benefits to sound localisation in some cases^4,7^. However, this is only possible for a small proportion of CI users (around 9%^8^) and residual hearing deteriorates at a faster rate after implantation^9^. Localisation can also be improved through the implantation of a second CI in the other ear^4,5^. However, this approach is expensive, poses a surgical risk, risks vestibular dysfunction and the loss of residual hearing, and limits access to future technologies and therapies. Our approach of using haptics could bring enhanced localisation to the majority of CI candidates who have severely limited localisation ability without the need for an expensive, invasive surgery to fit a second CI.

Haptic cues for spatial hearing have not previously been used to augment CI listening. However, historically, a small number of studies have looked at whether spatial cues can be provided through haptic stimulation on the upper arms^10^ or fingertips^11–14^ of young normal-hearing listeners. In 1955, Von Bekesy described subjective reports of people being able to learn to locate sounds with the upper arms^10^, and later studies using the fingertips provided further support for the idea that spatial hearing cues can be transferred through the skin^12,13^. Furthermore, recent work has shown that haptic stimulation can be used to enhance speech intelligibility in background noise for CI users^15–17^. Together, this research suggests that haptic stimulation may be able to augment the limited electrical signal from the implant to enhance CI spatial hearing.

In the current study, we investigated whether CI users’ ability to locate speech can be improved by augmenting the electrical signal provided by the implant with a haptic signal (electro-haptic stimulation^17^). We derived this haptic signal from the audio that would be received by CI or hearing aid microphones behind each ear. The haptic stimulus consisted of the amplitude envelope of the speech taken from bands in the frequency range where the ILD cues are largest (see Methods). The signal from each ear was then remapped to a frequency range where the skin is most sensitive to vibration and delivered to each wrist. This meant that the intensity difference between the wrists corresponded to the intensity difference between the ears. Our signal processing and haptic signal were designed to be readily deliverable by a low-latency wearable device with low power consumption.

We measured localisation ability under three conditions: audio only, combined audio and haptic (Audio-haptic), and haptic only. All conditions were measured before and after a short training regime (lasting around 15 minutes per condition). It was hypothesized that the haptic signal would allow participants to localise stimuli more accurately in the Audio-haptic condition than in the Audio-only condition. After training, it was anticipated that multisensory integration of the audio and haptic cues would occur, resulting in more accurate sound localisation in the Audio-haptic condition than in the Haptic-only condition.

## Results

**Figure 1:**
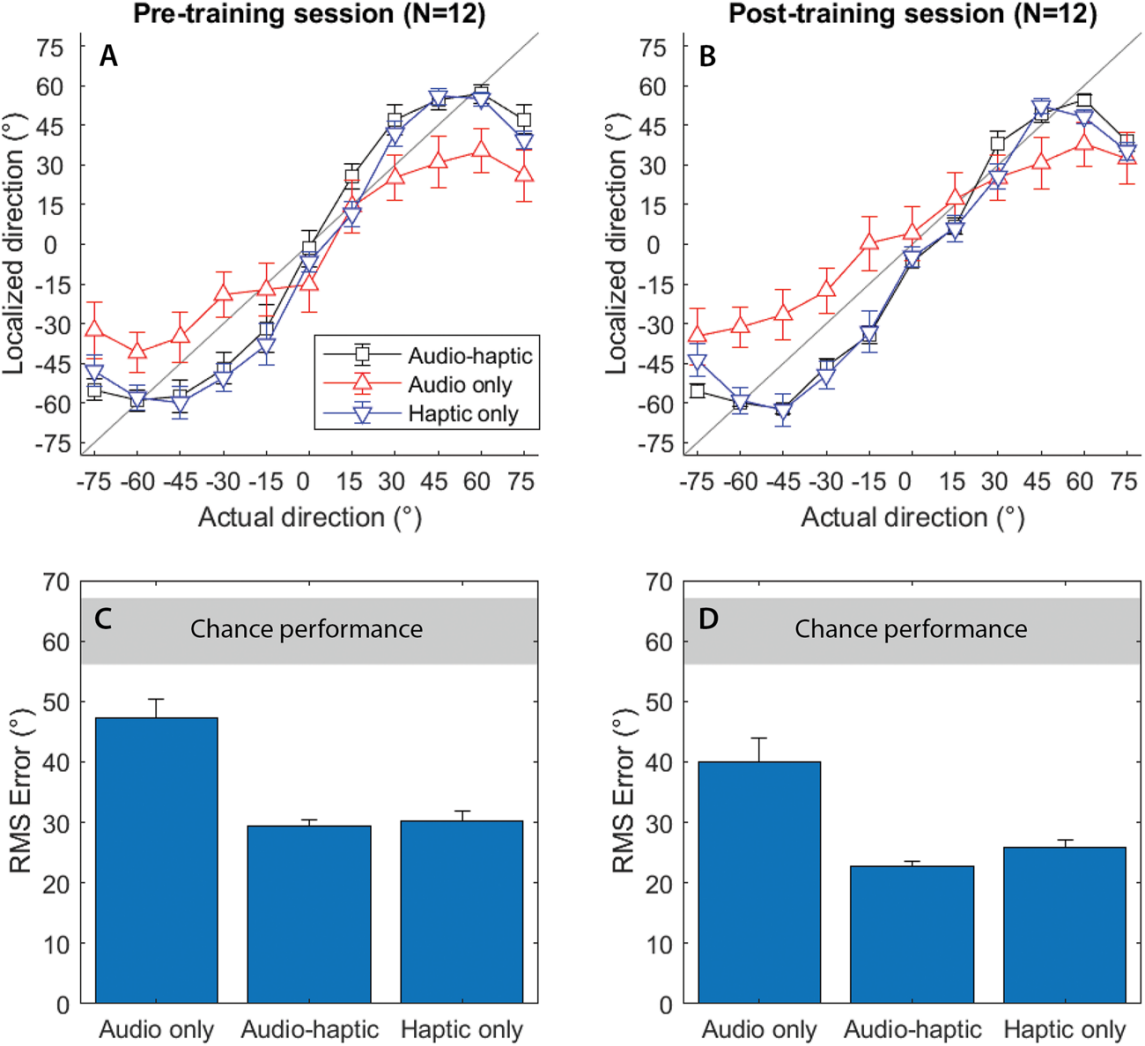
Haptic stimulation significantly reduces localisation error in cochlear implant users. (**A**,**B**) Mean response location vs actual sound source location before and after training (grey line = perfect localisation performance). (**C**,**D**) RMS error before and after training (grey bar = chance performance, +/− 95% confidence). Error bars show the standard error of the mean.

We tested twelve CI users’ ability to localise a speech stimulus in the horizontal plane, before and after a short training regime. Both unilateral CI users (who have a CI in one ear and no device in the other ear) and bimodal users (who have a CI in one ear and a hearing aid in the other ear) were tested in this study, which reflects the variety of implant and hearing aid configurations present in the population. Participants were tested using their everyday CI and hearing aid configuration to maximize ecological validity. Eleven loudspeakers were arranged in an arc around the participants from 75° to the left and right of centre. Participants were instructed to identify which loudspeaker the speech stimulus originated from. Figure 1 illustrates where participants perceived the speech to originate from compared to true location of the speech stimulus (upper panels), and shows localisation error in each of the three conditions, before and after training (lower panels).

**Figure 2:**
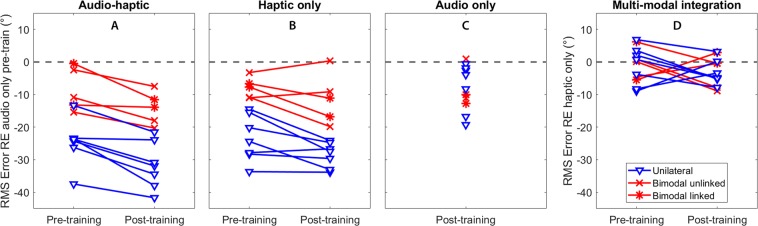
Training improves localisation performance and facilitates multi-modal integration. (**A**,**B**) Change in RMS error for each individual for the Audio-haptic and Haptic-only conditions relative to the Audio-only condition in the pre-training session. (**C**) Change in RMS error for the audio-only condition after training. (**D**) Performance in the Audio-haptic condition relative to the Haptic-only condition before and after training. Users with unilateral and bimodal device configurations with and without linked devices are indicated by different lines and markers (see legend).

We found that haptic stimulation enhanced localisation performance for CI users (*F*(1.2,12.3) = 25.3, *p* < 0.001, *η*_*p*_^2^ = 0.697). We also found that localisation performance improved between pre- and post-training testing sessions (*F*(1,11) = 36.5, *p* < 0.001, *η*_*p*_^2^ = 0.768). The interaction between these factors was non-significant (*F*(1.9,14.6) = 1.0, *p* = 0.37). We then investigated whether participants were able to utilize the additional spatial hearing cues available through the haptic signal to localise speech more accurately. We found that the root-mean-square (RMS) error was significantly lower in the Audio-haptic condition compared to the Audio-only condition both before training (*t*(11) = 5.9, *p* < 0.001, *d* = 1.69) and after training (*t*(11) = 4.3, *p* = 0.005, *d* = 1.24; all *t*-test *p*-values are corrected for multiple comparisons [see Methods]). Before training, RMS error reduced by 17.9°, from 47.2° to 29.3° on average (*SE* = 3.05). After training, RMS error reduced by 17.2°, from 39.9° to 22.7° on average (*SE* = 4.0). All participants performed better in the Audio-haptic condition than the Audio-only condition in both sessions (see Fig. 2), with the benefit ranging from a 0.5° (P7; bimodal linked; pre-training) to a 37.7° reduction in RMS error (P8; unilateral; post-training).

Next, we investigated whether completing a short training regime (lasting around 15 minutes per condition) would allow participants to improve their ability to localise sounds using combined audio and haptic stimulation. Performance in the Audio-haptic condition was found to be significantly better in the post-training session than in the pre-training session (*t*(11) = 5.8, *p* < 0.001, *d* = 1.68). With training, RMS error reduced by 6.6° in the Audio-haptic condition (from 29.3° to 22.7°; *SE* = 1.13). We also assessed whether completing the training regime allowed participants to integrate information from the audio and haptic stimulation to enhance localisation performance. There was no difference in performance between the Haptic-only and Audio-haptic conditions in the pre-training session (*p* = 0.566). However, in the post-training session, participants were able to locate sounds more accurately (a 3.1° enhancement) with Audio-haptic stimulation than with only haptic stimulation (*t*(11) = 2.6, *p* = 0.048, *d* = 0.66).

We found that even without audio cues, haptic stimulation could be used to determine spatial location. Localisation performance was better in the Haptic-only condition than in the Audio-only condition, with participants performing with a significantly smaller RMS error both before (30.2° vs 47.2°; *t*(11) = 6.00, *p* < 0.001, *d* = 0.740) and after training (25.9° vs 39.9°; *t*(11) = 3.89, *p* = 0.012, *d* = 1.123). We also observed that most participants were able to improve in the Audio-only condition between sessions, with RMS error reducing from an average of 47.2° to 39.9° (*SE* = 1.95; *t*(11) = 3.70, *p* = 0.012, *d* = 1.07).

One factor that may have affected performance in the task is the hearing device configurations that participants used. We measured performance in seven unilateral and five bimodal CI users. Two bimodal users were using a ‘linked’ configuration, in which a CI in one ear and a hearing aid in the other ear share audio processing to reduce distortion of spatial hearing cues. We observed that the participants with unilateral configurations had poorer performance with audio cues alone than bimodal users (54.3° and 37.2° respectively before training; *t*(10) = 4.18, *p* = 0.008, *d* = 2.44). Both groups reached a similar level of performance with audio and haptic stimulation combined (22.6° and 23.0° respectively). As such, unilateral users had a greater enhancement in performance when haptic stimulation was combined with audio than bimodal users (see Fig. 2) in both the pre-training (24.6° vs 8.5°; *t*(10) = 3.99, *p* = 0.009, *d* = 2.35) and post-training (24.1° vs 7.5°; *t*(10) = 2.48, *p* = 0.034, *d* = 1.52) sessions. They also had a significantly greater performance enhancement in the Haptic-only condition than bimodal users in both the pre-training (*t*(10) = 4.54, *p* = 0.005, *d* = 2.83) and post-training sessions (*t*(10) = 2.85, *p* = 0.034, *d* = 1.68).

## Discussion

The vast majority of CI users are implanted in only one ear and are very poor at locating sounds. In this study, we found that sound localisation accuracy improved substantially when audio and haptic stimulation were provided together (electro-haptic stimulation). Even with no training, adding haptic stimulation reduced the RMS error from 47.2° to 29.3° on average. This performance is similar to the average performance achieved by CI users with implants in both ears (~27°)^4,18^, or users with a CI in one ear and healthy hearing in the other (~28°)^4^. After a short training regime, participants’ average RMS error with electro-haptic stimulation was reduced to just 22.7°, which is comparable to the performance of bilateral hearing aid users (~19°)^4,19^. These results suggest that haptic stimulation can be used to substantially improve localisation for CI users with one implant, without the need for expensive and invasive surgery to fit a second implant.

The size of the improvement given by adding haptic stimulation depended on participants’ hearing device configuration. Participant’s with a unilateral configuration had poorer localisation with audio only than bimodal users (54.3° and 37.2° respectively, before training), which is consistent with previous studies^4^ and the fact that bimodal users are likely to have better access to spatial hearing cues. Despite this difference with audio only, both groups reached a similar level of performance with electro-haptic stimulation (22.6° and 23° after training, respectively). Therefore, electro-haptic stimulation appears to give the largest gains in performance for CI users who struggle most with audio alone. Remarkably, four out of seven unilateral participants performed more than 30° better with electro-haptic stimulation than with audio only, after training. These large effects are particularly encouraging given that there is no established alternative approach for improving localisation in CI users with a single device.

Importantly, a short training regime allowed participants to effectively combine audio and haptic input. We found that, after training, our participants performed better with electro-haptic stimulation than with either audio only (17.2° better) or haptic stimulation only (3.1° better). In this study, both the audio and haptic signals were speech stimuli consisting of temporally complex amplitude modulations, rather than more simple stimuli, such as tones or noises. Recent work has provided strong evidence of the importance of the correlation of temporal properties for maximizing multisensory integration, and the advantage of these temporal properties being complex^20–24^. Therefore, our use of temporally complex stimuli may have facilitated effective integration of audio and haptic signals.

The audio-haptic enhancement in performance observed in the current study may be expected based on previous psychophysical, physiological, and anatomical findings. Psychophysicists have shown both that auditory stimuli can affect the perception of haptic stimuli^25–28^ and that haptic stimuli can affect the perception of auditory stimuli^29^. Multisensory interactions have also been shown in the core auditory cortices of ferrets, where substantial populations of neurons that respond to auditory stimulation are modulated by tactile stimulation^30^. Furthermore, anatomical studies have shown the convergence of somatosensory input at many stages along the ascending auditory pathway, from the cochlear nucleus (the first node in the ascending auditory pathway) to core auditory cortices^30–39^. Collectively, these studies provide compelling evidence of strong links between audition and touch and offer a neural basis for our finding that information from auditory and haptic stimulation can be effectively combined to improve behavioural performance.

In this study, like in many of the most effective haptic aids^40^, haptic stimulation was applied to the wrists. The wrist was selected as a practical candidate site for real-world use because wrist-worn devices do not typically impede everyday tasks and are easy to self-fit. Preserving the perceived intensity differences across the wrists is critical for this application, and additional testing is required to establish whether this would be affected by frequent changes in the relative positions of the wrists in everyday life. Encouragingly, researchers who found that haptic stimulation on one hand modulates haptic intensity perception on the other hand, found that this intensity modulation was not dependent on the relative hand positions^41^. However, there is a well-established effect of hand-crossing on temporal order judgement thresholds, with thresholds increasing substantially when the hands are crossed^42,43^. If required, candidate alternative sites might include the upper arms or upper forearms, which retain much of the convenience of the wrist but reduce the relative motion of the stimulation sites.

In the current study, less than one hour of training was provided. Despite this relatively small amount of training, we observed improvements in performance in all conditions (Audio-haptic, Haptic-only, and Audio-only). Future work should assess how generalizable training is to real-world listening and establish the optimum training regime to maximise audio-haptic performance. In this study, some of the observed performance improvement may have been due to participants learning to use spatial cues relating only to the specific loudspeaker positions used. However, previous work suggests that subjects can become more sensitive to spatial hearing cues with training^44^, indicating that our improvement in performance may be generalizable beyond the experimental procedure. Previous research has also shown that participants continue to improve their ability to identify speech presented through haptic stimulation after many hours of training^45–47^. This suggests that long-term training may give further improvement in haptic performance. Finally, haptic stimulation has been shown to support lip-reading after extensive training^48^, suggesting that long-term training may increase multisensory integration of audio and haptic inputs.

It is important to note that in the current study, performance was assessed under simplified acoustic conditions where participants identified the location of a single speech stimulus. Future work should investigate the benefits of electro-haptic stimulation in more complex acoustic environments, with multiple simultaneous sound sources. In such environments, it may be possible to improve performance through the use of algorithms that magnify spatial hearing cues, aid the segregation of multiple sounds, and reduce background noise^49–51^.

In this study, we showed that providing spatial information to CI users through haptic stimulation of the wrists substantially improves localisation. Our approach was designed to be easily transferable to a real-world application. The haptic signal was processed using a computationally lightweight algorithm that could be applied in real-time and was delivered at a vibration intensity that could readily be achieved by a low-cost wearable device. This could have an important clinical impact, providing an inexpensive, non-invasive means to dramatically improve spatial hearing in CI users.

## Methods

### Participants

Twelve CI users (4 male, 8 female; mean age = 52.6 years old, ranging from 41 to 63 years old) were recruited through the University of Southampton Auditory Implant Service. All participants were native British English speakers, had been implanted at least 6 months prior to the experiment, and had the capacity to give informed consent. Participants completed a screening questionnaire, confirming that they had no medical conditions and were taking no medication that may affect their sense of touch. Table 1 details the characteristics of the participants who took part in the study. Participants were instructed to use their normal hearing set up and not to adjust their settings during the experiment, and included seven unilateral users (a single implant), and five bimodal users (an implant and a contralateral hearing aid). One participant (P2) was categorized as having some residual hearing, defined here as having unaided thresholds at 250 and 500 Hz that are 65 dB HL or better in both ears.

**Table 1:** Summary of participant characteristics. *CI* = Cochlear implant, *HA* = Hearing aid.

Participant	Gender	Age	Device Left	Device Right	Years since implantation
1	M	59	CI: Cochlear CP920	HA: ReSound	3.2
2	F	42	CI: Cochlear CP920	None	4.4
3	F	54	None	CI: MED-EL Rondo	4.3
4	F	50	HA: Danalogic	CI: Cochlear CP1000	5.6
5	M	44	CI: Advanced Bionics Nadia Q70	HA: Phonak [linked]	1.0
6	F	49	CI: Cochlear CP 1000	HA: Oticon	1.5
7	M	58	HA: Phonak [linked]	CI: Advanced Bionics Naida Q90	0.6
8	F	41	None	CI: Cochlear CP 1000	10.6
9	F	61	CI: Med-El Sonnet	None	2.4
10	M	63	None	CI: Advanced Bionics Q90	0.7
11	F	58	CI: Advanced Bionics Naida Q70	None	9.1
12	F	52	CI: Advanced Bionics Naida Q70	None	11.3

Vibrotactile detection thresholds were measured at the fingertip and wrist at 31.5 Hz and 125 Hz following conditions and criteria specified in ISO 13091-1:2001^52^. One participant (P7) had elevated thresholds at the fingertips of the left and right index fingers at 125 Hz (1.8 and 1.0 ms^−2^, respectively). All others had vibrotactile detection thresholds within the normal range (<0.4 ms^−2^ RMS at 31.5 Hz, and <0.7 ms^−2^ RMS at 125 Hz^52^). The mean vibrotactile detection threshold at the skin of the wrist at 31.5 Hz was 0.65 ms^−2^ RMS, and at 125 Hz was 0.75 ms^−2^ RMS (averaged across left and right wrists; there are no published standards for normal wrist sensitivity).

### Stimuli

The speech stimulus consisted of recording of a female voice saying “*Where am I speaking from?*”, recorded using a Rode M5 microphone in the small anechoic chamber at the Institute of Sound and Vibration Research (ISVR), UK. This audio file is available at: 10.5258/SOTON/D1206. The speech signal was presented at a level of 65 dB SPL LAeq. The intensity of each presentation was roved randomly +/− 2.5 dB around 65 dB SPL to prevent participants learning to locate the speech based on absolute level cues. Each loudspeaker was calibrated at the listening position using a Brüel & Kjær (B&K) G4 type 2250 sound level meter (which was calibrated using a B&K type 4231 sound calibrator).

For the haptic signal, head-related transfer functions (HRTFs) were taken from The Oldenburg Hearing Device HRTF Database^53^ and applied to the speech signal separately for each loudspeaker position used in the experiment. The three-microphone behind the ear (“BTE_MultiCh”) HRTFs were used, in order to match a typical CI signal. The signal was then downsampled to a sampling frequency of 22050 Hz. Each channel of this stereo signal was then passed through an FIR filter bank with four frequency channels with center frequencies equally spaced on the ERB scale^54^. The edges of the bands were between 1,000 and 10,000 Hz, a frequency range that contains the most speech energy^55^ and large ILDs^56^. The Hilbert envelope for each frequency channel was calculated and a first-order low-pass filter was applied with a cut-off frequency of 10 Hz to extract the speech envelope. This low-pass filter emphasised the modulation frequency range between around 1 and 30 Hz, which is the most important range for speech intelligibility^57^. These signals were then used to modulate the amplitude envelopes of four fixed-phase tonal carriers with center frequencies of 50, 110, 170, and 230 Hz. This frequency range was selected because it is one to which the tactile system is highly sensitive^58^. The carriers had a 60 Hz frequency spacing and fixed phases. These carriers were chosen because they would be expected to be individually discriminable based on estimates of vibrotactile frequency difference limens^59^. These were then summed and presented via the *HVLab* tactile vibrometer. This signal processing strategy was similar to that used in Fletcher *et al*.^17^. Haptic stimuli were presented at a maximum acceleration magnitude of 1.84 ms^−2^ RMS (e.g. the left vibrometer when the signal is presented 75° to the left). The intensity difference between the two shakers directly corresponded to the intensity difference between the ears extracted from the HRTF, with no additional scaling applied.

The vibrometers were calibrated using a B&K type 4294 calibration exciter. During piloting of the experiment, waveforms from the shakers were recorded using the PCB Piezotronics ICP 353B43 accelerometers built into the HVLab tactile vibrometers, and visually inspected to ensure that the signals were faithfully reproduced.

### Apparatus

**Figure 3:**
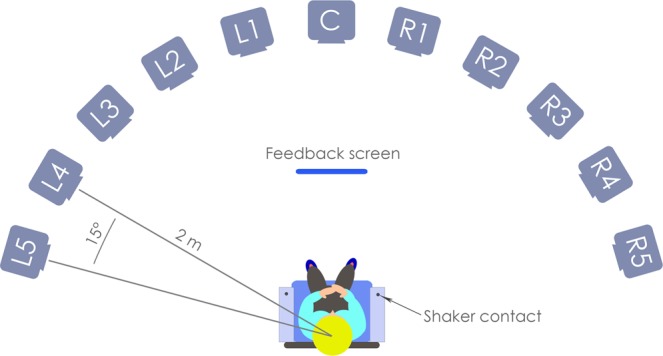
Schematic illustration of the experimental set up. This schematic shows the audio-only condition, where the participant has their hands in their lap rather than their wrists on the shaker contacts. On each trial the audio stimulus was presented through one of the 11 loudspeakers, positioned at points between 75° to the left and 75° to the right of the centre.

Participants were seated in the centre of the ISVR small anechoic chamber. Eleven Genelec 8020 C PM Bi-Amplified Monitor System loudspeakers were positioned in an arc in front of the participant, from −75° to 75°, with 15° spacing between the loudspeakers (see Fig. 3). The speakers were placed 2 m from the centre of the participant’s head, at approximately the same height as their ears in a sitting position (1.16 m). The speakers were labelled L5 through R5 as illustrated in Figure 3. An acoustically treated 20′′ wide-screen monitor for displaying feedback and giving instructions was positioned on the floor 1 m in front of the participant. Two *HVLab* tactile vibrometers were placed beside the participant’s chair and were used to deliver the vibrotactile signal to the participants’ wrists (the palmer surface of the distal forearm) via a rigid 10-mm nylon probe with no surround to maximise the area of skin excitation. All stimuli were controlled using a custom MATLAB script (MATLAB 2018b) via a RME M-32 DA 32-Channel digital to analog converter.

During testing, the experimenter sat in a separate control room. The participants’ verbal responses were monitored using a Shure BG 2.1 dynamic microphone placed low behind the participant’s seat, amplified by a Creek OBH-21 headphone amplifier, and played back through a pair of Sennheiser HD 380 Pro headphones. Participants were monitored visually using a Microsoft HD-3000 webcam.

### Procedure

The experiment was conducted over two sessions not more than 5 days apart (average number of days = 1.58, *SE* = 0.38). In session 1, the participant first filled out a health questionnaire^16^ and had their vibrotactile thresholds measured following conditions and criteria specified in ISO 13091-1:2001^52^. The task was then demonstrated to the participant by presenting the speech stimulus from speakers C (centre), L5 (75° left), and R5 (75° right). This demonstration was repeated for each of three conditions: Audio only, combined audio and haptic stimulation, and haptic stimulation only. At this stage, it was confirmed that the speech stimuli were clearly audible, and participants were given the opportunity to ask any questions.

A testing block of was then conducted, lasting around 20–25 minutes. In each trial, the participant was instructed to fixate on the central speaker (marked with a red cross), and to keep their head still. The speech stimulus was presented from one of the 11 loudspeakers, and the participant’s task was to identify which loudspeaker was the source. For each condition, the stimulus was presented from each speaker in a randomised order. This procedure was then repeated four times. Localisation accuracy was calculated as RMS error using the *D* statistic described by Rakerd and Hartman^60^. Chance performance level was estimated using a Monte Carlo simulation with 100,000 samples, assuming unbiased responses.

Responses were made verbally and recorded in the control room by the experimenter, who was blinded to the true source of the stimulus. The participant was monitored via webcam, to ensure that they did not move their head, were using the vibrometers in the haptic stimulation conditions, and were not making contact with the vibrometers in the audio only condition. The vibrometers were near silent, but were left on in all conditions to control for any subtle audio cues.

After a break of at least 15 minutes, the participant completed a training block, which was the same as the testing block except that stimuli were presented in a new randomised order and performance feedback was provided on the screen. The screen displayed an illustration of the speaker array (similar to Fig. 3). If the participant was correct, an illustration of the target speaker lit up green. If the participant was incorrect, an illustration of the chosen speaker lit up red, and the target speaker lit up green. In the second session, the participant completed a further training block, followed by a final testing block.

The experimental protocol was approved by the University of Southampton Ethics Committee (ERGO ID: 46201) and the UK National Health Service Research Ethics Service (Integrated Research Application System ID: 256879). All research was performed in accordance with the relevant guidelines and regulations.

### Statistics

Performance was calculated as RMS error from the target location in degrees arc for all trials in each condition within a session^60^. Primary analysis of performance on the spatial hearing task consisted of a 3 × 2 repeated measures analysis of variance (ANOVA) with factors ‘Condition’ (Audio-only, Audio-haptic, or Haptic-only) and ‘Session’ (before or after training). Mauchly’s test indicated that the assumption of sphericity had been violated (*χ*^2^(2) = 15.5, *p* < 0.001), so degrees of freedom were corrected using Greenhouse-Geisser estimates of sphericity (*ε* = 0.56). The ANOVA used an alpha level of 0.05. Post-hoc two-tailed *t*-tests were conducted to investigate these effects. Nine two-tailed paired-samples *t*-tests (with a Bonferroni-Holm correction for multiple comparisons) were used to investigate performance across the three conditions and two sessions. Five two-tailed independent samples *t*-tests (also with a Bonferroni-Holm correction) were conducted to analyse differences in performance between the seven unilateral and five bimodal CI users who took part in the study.

## Data Availability

The dataset and stimuli from the current study is publicly available through the University of Southampton’s Research Data Management Repository at: 10.5258/SOTON/D1206.

## References

[1] Wilson BS (2015). Getting a decent (but sparse) signal to the brain for users of cochlear implants. Hearing research.

[2] Spriet A (2007). Speech Understanding in Background Noise with the Two-Microphone Adaptive Beamformer BEAM in the Nucleus Freedom Cochlear Implant System. Ear and Hearing.

[3] McDermott HJ (2004). Music Perception with Cochlear Implants: A Review. Trends in Amplification.

[4] Dorman MF, Loiselle LH, Cook SJ, Yost WA, Gifford RH (2016). Sound Source Localization by Normal-Hearing Listeners, Hearing-Impaired Listeners and Cochlear Implant Listeners. Audiol. Neurootol..

[5] Verschuur CA, Lutman ME, Ramsden R, Greenham P, O’Driscoll M (2005). Auditory Localization Abilities in Bilateral Cochlear Implant Recipients. Otology & Neurotology.

[6] Peters BR, Wyss J, Manrique M (2010). Worldwide trends in bilateral cochlear implantation. The Laryngoscope.

[7] O’Connell, B. P., Dedmon, M. M. & Haynes, D. S. Hearing Preservation Cochlear Implantation: a Review of Audiologic Benefits, Surgical Success Rates, and Variables That Impact Success. *Curr. Otorhinolaryngol. Rep.* **5**, 286–294 (2017).

[8] Verschuur C, Hellier W, Teo C (2016). An evaluation of hearing preservation outcomes in routine cochlear implant care: Implications for candidacy. Cochlear Implants International.

[9] Wanna GB (2018). Predictive factors for short- and long term hearing preservation in cochlear implantation with conventional length electrodes. Laryngoscope.

[10] Békésy GV (1955). Human skin perception of traveling waves similar to those on the cochlea. The Journal of the Acoustical Society of America.

[11] Gescheider GA (1970). Some comparisons between touch and hearing. IEEE Transactions on Man-Machine Systems.

[12] Frost BJ, Richardson BL (1976). Tactile localization of sounds: Acuity, tracking moving sources, and selective attention. The Journal of the Acoustical Society of America.

[13] Richardson BL, Frost BJ (1979). Tactile localization of the direction and distance of sounds. Perception & Psychophysics.

[14] Richardson BL, Wuillemin DB, Saunders FJ (1978). Tactile discrimination of competing sounds. Perception & Psychophysics.

[15] Huang J, Sheffield B, Lin P, Zeng F-G (2017). Electro-Tactile Stimulation Enhances Cochlear Implant Speech Recognition in Noise. Scientific Reports.

[16] Fletcher, M. D., Mills, S. R. & Goehring, T. Vibro-Tactile Enhancement of Speech Intelligibility in Multi-talker Noise for Simulated Cochlear Implant Listening. *Trends in Hearing* **22** (2018). 10.1177/2331216518797838 PMC6144588 30222089

[17] Fletcher MD, Hadeedi A, Goehring T, Mills SR (2019). Electro-haptic hearing: Speech-in-noise performance in cochlear implant users is enhanced by tactile stimulation of the wrists. Scientific Reports.

[18] Aronoff JM (2010). The use of interaural time and level difference cues by bilateral cochlear implant users. The Journal of the Acoustical Society of America.

[19] Dunn, C. C., Perreau, A., Gantz, B. & Tyler, R. S. Benefits of Localization and Speech Perception with Multiple Noise Sources in Listeners with a Short-Electrode Cochlear Implant. *J. Am Acad. Audiol.* **21**, 44–51 (2010). 10.3766/jaaa.21.1.6 PMC2809934 20085199

[20] Ernst MO, Bülthoff HH (2004). Merging the senses into a robust percept. Trends in cognitive sciences.

[21] Parise CV, Spence C, Ernst MO (2012). When correlation implies causation in multisensory integration. Current Biology.

[22] Fujisaki W, Nishida S (2005). Temporal frequency characteristics of synchrony–asynchrony discrimination of audio-visual signals. Experimental Brain Research.

[23] Burr D, Silva O, Cicchini GM, Banks MS, Morrone MC (2009). Temporal mechanisms of multimodal binding. Proceedings of the Royal Society B: Biological Sciences.

[24] Parise CV, Ernst MO (2016). Correlation detection as a general mechanism for multisensory integration. Nature communications.

[25] Jousmäki V, Hari R (1998). Parchment-skin illusion: sound-biased touch. Current biology.

[26] Yau JM, Weber AI, Bensmaia S (2010). Separate mechanisms for audio-tactile pitch and loudness interactions. Frontiers in Psychology.

[27] Yau JM, Olenczak JB, Dammann JF, Bensmaia SJ (2009). Temporal Frequency Channels Are Linked across Audition and Touch. Current Biology.

[28] Crommett LE, Pérez-Bellido A, Yau JM (2017). Auditory adaptation improves tactile frequency perception. Journal of Neurophysiology.

[29] Gick B (2009). & Derrick, D. Aero-tactile integration in speech perception. Nature.

[30] Meredith MA, Allman BL (2015). Single-unit analysis of somatosensory processing in the core auditory cortex of hearing ferrets. European Journal of Neuroscience.

[31] Shore SE, Vass Z, Wys NL, Altschuler RA (2000). Trigeminal ganglion innervates the auditory brainstem. Journal of Comparative Neurology.

[32] Aitkin LM, Kenyon CE, Philpott P (1981). The representation of the auditory and somatosensory systems in the external nucleus of the cat inferior colliculus. Journal of Comparative Neurology.

[33] Shore SE, El Kashlan H, Lu J (2003). Effects of trigeminal ganglion stimulation on unit activity of ventral cochlear nucleus neurons. Neuroscience.

[34] Allman BL, Keniston LP, Meredith MA (2009). Adult deafness induces somatosensory conversion of ferret auditory cortex. Proceedings of the National Academy of Sciences.

[35] Meredith MA, Keniston LP, Allman BL (2012). Multisensory dysfunction accompanies crossmodal plasticity following adult hearing impairment. Neuroscience.

[36] Kanold PO, Davis KA, Young ED (2010). Somatosensory context alters auditory responses in the cochlear nucleus. Journal of neurophysiology.

[37] Foxe JJ (2000). Multisensory auditory–somatosensory interactions in early cortical processing revealed by high-density electrical mapping. Cognitive Brain Research.

[38] Gobbelé R (2003). Activation of the human posterior parietal and temporoparietal cortices during audiotactile interaction. NeuroImage.

[39] Caetano G, Jousmäki V (2006). Evidence of vibrotactile input to human auditory cortex. NeuroImage.

[40] Thornton R. D. & Phillips, A. J. A comparative trial of four vibrotactile aids. In *Tactile Aids for the Hearing Impaired*, edited by I. R. Summers Whurr, London, pp. 231–251 (1992).

[41] Rahman S, Yau JM (2019). Somatosensory interactions reveal feature-dependent computations. J. Neurophysiol..

[42] Yamamoto S, Kitazawa S (2001). Reversal of subjective temporal order due to arm crossing’. Nature Neuroscience.

[43] Shore D, Spry E, Spence C (2002). Confusing the mind by crossing the hands. Cognitive Brain Research.

[44] Wright BA, Fitzgerald MB (2001). Different patterns of human discrimination learning for two interaural cues to sound-source location. Proceedings of the National Academy of Sciences.

[45] Weisenberger JM (1986). Sensitivity to amplitude‐modulated vibrotactile signals. The Journal of the Acoustical Society of America.

[46] Saunders, F., Hill, W. & Simpson, C. Speech perception via the tactile mode: Progress report. In *ICASSP’76*. *IEEE International Conference on Acoustics*, *Speech*, *and Signal Processing* **1**, 594–597 (IEEE, 1976).

[47] Sparks DW, Kuhl PK, Edmonds AE, Gray GP (1978). Investigating the MESA (Multipoint Electrotactile Speech Aid): The transmission of segmental features of speech. The Journal of the Acoustical Society of America.

[48] Kishon-Rabin L, Boothroyd A, Hanin L (1996). Speechreading enhancement: A comparison of spatial-tactile display of voice fundamental frequency (F 0) with auditory F 0. The Journal of the Acoustical Society of America.

[49] Moore BCJ, Kolarik A, Stone MA, Lee Y-W (2016). Evaluation of a method for enhancing interaural level differences at low frequencies. The Journal of the Acoustical Society of America.

[50] Pirhosseinloo, S. & Kokkinakis, K. An Interaural Magnification Algorithm for Enhancement of Naturally-Occurring Level Differences. *Interspeech*, 2558–2561 (2016).

[51] Williges, B., Jürgens, T., Hu, H. & Dietz, M. Coherent Coding of Enhanced Interaural Cues Improves Sound Localization in Noise With Bilateral Cochlear Implants. *Trends in Hearing* **22** (2018). 10.1177/2331216518781746 PMC6048749 29956589

[52] International Standards Organisation. ISO 13091-1:2001 - Mechanical vibration–Vibrotactile perception thresholds for the assessment of nerve dysfunction–Part 1: Methods of measurement at the fingertips. *ISO* Available at, http://www.iso.org/iso/catalogue_detail.htm?csnumber=32509. (Accessed: 21st July 2016) (2001).

[53] Denk, F., Ernst, S. M., Ewert, S. D. & Kollmeier, B. Adapting hearing devices to the individual ear acoustics: Database and target response correction functions for various device styles. *Trends in hearing* **22** (2018). 10.1177/2331216518779313 PMC5992802 29877161

[54] Glasberg BR, Moore BC (1990). Derivation of auditory filter shapes from notched-noise data. Hear. Res..

[55] Byrne D (1994). An international comparison of long‐term average speech spectra. The Journal of the Acoustical Society of America.

[56] Feddersen WE, Sandel TT, Teas DC, Jeffress LA (1957). Localization of High‐Frequency Tones. The Journal of the Acoustical Society of America.

[57] Drullman R, Festen J, Plomp R (1994). Effect of temporal envelope smearing on speech reception. The Journal of the Acoustical Society of America.

[58] Verrillo RT (1963). Effect of contactor area on the vibrotactile threshold. The Journal of the Acoustical Society of America.

[59] Rothenberg M, Verrillo R, Zahorian S, Brachman M, Bolanowski S (1977). Vibrotactile frequency for encoding a speech parameter. The Journal of the Acoustical Society of America.

[60] Rakerd B, Hartmann WM (1986). Localization of sound in rooms, III: Onset and duration effects. The Journal of the Acoustical Society of America.

